# Physiological Assessment of Water Stress in Potato Using Spectral Information

**DOI:** 10.3389/fpls.2017.01608

**Published:** 2017-09-20

**Authors:** Angela P. Romero, Andrés Alarcón, Raúl I. Valbuena, Carlos H. Galeano

**Affiliations:** Centro de Investigación Tibaitatá, Corporación Colombiana de Investigación Agropecuaria (CORPOICA) Mosquera, Colombia

**Keywords:** drought tolerance, yield, potato breeding, reflectance indexes, correlation, stomatal conductance

## Abstract

Water stress in potato (*Solanum tuberosum* L.) causes considerable losses in yield, and therefore, potato is often considered to be a drought sensitive crop. Identification of water deficit tolerant potato genotypes is an adaptation strategy to mitigate the climatic changes that are occurring in the Cundiboyacense region in Colombia. Previous studies have evaluated potato plants under water stress conditions using physiological analyses. However, these methodologies require considerable amounts of time and plant material to perform these measurements. This study evaluated and compared the physiological and spectral traits between two genotypes, Diacol Capiro and Perla Negra under two drought levels (10 and 15 days without irrigation from flowering). Reflectance information was used to calculate indexes which were associated with the physiological behavior in plants. The results showed that spectral information was correlated (ρ < 0.0001) with physiological variables such as foliar area (FA), total water content (H_2_Ot), relative growth rate of potato tubers (RGTtub), leaf area ratio (LAR), and foliar area index (AFI). In general, there was a higher concentration of chlorophyll under drought treatments. In addition, Perla Negra under water deficit treatments did not show significant differences in its physiological variables. Therefore, it could be considered a drought tolerant genotype because its physiological performance was not affected under water stress conditions. However, yield was affected in both genotypes after being subject to 15 days of drought. The results suggested that reflectance indexes are a useful and affordable approach for potato phenotyping to select parent and segregant populations in breeding programs.

## Introduction

Potato (*Solanum tuberosum* L.) is the fourth most important food crop in the world (FAO, 2011). It plays an important role as a food supply in developing countries because it is an affordable and rich source of carbohydrates. In Colombia, the average potato *per capita* consumption is more than 62 kg/year, so it is a fundamental diet element for the population. The country produces between 1.8 and 2 million tons of potato per year with an average yield of 19 ton/ha (DANE-ENEA, 2014). However, the demand for potato is growing for both fresh and processed markets. Therefore, breeders have several challenges to address such as finding high-yielding potato cultivars adapted to climate change; currently in the country‘s potato growing areas extreme weather events like droughts are becoming more frequent and are causing greater crop losses. The effects of climate change will be particularly pronounced in lower-latitude regions where most developing countries are located. In Africa, Asia and Latin America for instance, yields could decline between 20 and 40% in the future if no effective adaptation measures are taken (FAO, 2011). Specifically in Colombia, a good portion of the agro-ecosystems suitable for potato production are vulnerable to increased aridity, soil erosion, desertification, and variations in the hydrological system as consequence of climate change (United Nations Development Programme (UNDP), 2010). Therefore, potato breeders have to find new cultivars resistant to both biotic and abiotic stresses and, at the same time, they must guarantee that these possess high yielding and good tuber qualities, and with good producer, market and consumer acceptance.

There are many physiological variables used to evaluated water stress in plants. For instance, gas exchange is the method that most researchers use to study drought responses. This approach is based on the fact that drought stress reduces stomatal conductance in potato when leaf water potential values fall below −0.6 MPa (Ahmadi et al., 2010). In addition, a range of low photosynthetic rate values between 1 and 3.6 μCO_2_ m^−2^ s^−1^ have been used to describe water stress conditions in potato grown in greenhouses under natural light (Vasquez-Robinet et al., 2008). Similarly, in well tuberized potatoes the response of the photosynthetic rate to drought depends on mesophyll conductance (Schapendonk et al., 1989), relative water content (RWC) and light intensity (Basu et al., 1999).

On the other hand, canopy spectral reflectance is based on the differential pattern of light reflectance on leaves at photosynthetically active (400–700 nm) and infrared (700–1,000 nm) wavelengths (Bowman et al., 2015). Additionally, much more specific narrow-band regions such as the red edge (maximum slope of vegetation reflectance from 690 to 740 nm) for predicting plant stress have been used (Clay et al., 2006). Thus, spectral vegetation indexes were designed to evaluate vegetation condition, foliage, cover, phenology and drought monitoring (Padilla et al., 2011), as well as leaf nitrogen (N) (Pilon et al., 2010), photosynthetic characteristics (Aparicio et al., 1999), chlorophyll content and plant water status (Kokaly et al., 2003; Rosso et al., 2005; Gutierrez et al., 2010). Although attempts to develop indexes based on a mathematical function of the amount of reflected light at various wavelengths to be used as a water status proxy have been successfully applied (Zygielbaum, 2009), it is necessary to conduct more studies to standardize the use of this technique for each specific crop and type of stress.

Vegetation indexes have widely been reviewed in literature (Milton et al., 2007; Ahmad, 2012). The ones that need to be highlighted are the normalized vegetation index (NDVI) that is associated with canopy density (i.e., leaf area or percent cover) or total biomass (Verhulst and Govaerts, 2010), the simple ratio (SR) that is related to changes in the amount of green biomass and pigment content, i.e., high values for healthy vegetation and low ones for soil, water, etc. (Tucker and Sellers, 1986). The photochemical reflectance index (PRI) measures the xanthophyll cycle activity which is related to photosynthetic light-use efficiency in plants (Coops et al., 2010). Studies have demonstrated that the PRI is a physiological index sensitive to the epoxidation state of the xanthophyll cycle pigments and to photosynthetic efficiency, serving as a proxy for short-term changes in photosynthetic activity, stress conditions, and pigment absorption, but is highly affected by illumination conditions, viewing geometry and canopy structure (Hernández-Clemente et al., 2011). The pigment-specific simple ratio of chlorophyll-a (PSSRa) measures chlorophyll-a content and has been used for estimating vegetative biomass (Babar et al., 2006b). The reflectance water index (WI) is defined as the ratio between the reflectance at a water band wavelength (900 nm) to a reflectance wavelength where there is no absorption due to water content variability (970 nm) (Prasad et al., 2007). The normalized water index 1 (NWI1), the normalized water index 2 (NWI2), the normalized water index 3 (NWI3) and the normalized water index 4 (NWI4) are an alternative approach to select high yielding lines for diverse environments (Babar et al., 2006b; Prasad et al., 2007). The previous four indexes are defined as the Oryza N index (ONI) and they are useful for the nondestructive monitoring and diagnosis of plant N status. Lastly, the dry Zea N index (DZNI) is used for the early detection of N deficiency in plants (Bowman et al., 2015). Specifically in potato, spectral information has been used to identify N rate (Booij and Uenk, 2004; Bowen et al., 2005; Van Evert et al., 2012) and water content (Elbatawi et al., 2008). Additionally, near-infrared reflectance was used to estimate the proportion of ground covered by potato canopy (Bouman et al., 1992).

Regarding drought stress information high phenotyping costs to get accurate data has been a huge bottleneck. Therefore, spectral-related data have instead been used to calculate vegetation indexes that have shown correlation to plant growth, yield and early detection of biotic or abiotic stress in plants, allowing the early and quick evaluation of a wide variety of cultivars. Spectroscopy in particular, has the capacity to detect different materials by discriminating their reflectance information. For instance in plant physiology, reflectance information has been widely used to determine plant health, water content, environmental stress, and other agronomic traits (Ceccato et al., 2001; Campbell et al., 2007; Nijland et al., 2014).

It is well known that drought limits crop plants‘ productivity by affecting photosynthetic processes at the canopy, leaf or chloroplast levels, either directly or by feedback inhibition if photosynthetic transport to sink organs is limited (Jones and Corlett, 1992). Baracaldo et al. (2014) showed that when plants suffered transpiration reduction, some adaptations to abiotic stress were among others, leaf yellowing, epinasty and abscission. Tuber production generally correlates with the plant‘s available water content; however, some genotypes produce higher yields than others under limited water supply. Considerable variation in drought tolerance has been found in potato varieties, elite clones, landraces and wild relatives (Schafleitner et al., 2007; Coleman, 2008). Vasquez-Robinet et al. (2008) found that individuals of *S. tuberosum* spp. *andigena* showed more drought tolerance compared to individuals of *S. tuberosum* spp. *tuberosum*, and this has been attributed to the induction of heat shock proteins and antioxidant genes encoding proteins in the chloroplast, as well as genes for anthocyanin synthesis and transport that could be important in case of repeated stress. These findings confirm the putative roll of anthocyanins and their importance in ameliorating environmental stresses induced by drought (Chalker-Scott, 1999).

Furthermore, drought phenotyping studies in potato have been carried out using biomass traits, yield components and physiological indexes (Saravia et al., 2016). However, as these evaluations involved destructive sampling and considerable amount of labor, it is necessary to find an affordable and accurate alternative to such methods, as spectral reflectance to evaluate water stress in plants. Still, the use of high-throughput phenotypic testing, specifically spectral reflectance, has been limited (Dammer et al., 2016). Therefore, the aims of this study are: (1) to evaluate spectral reflectance indexes for potato drought evaluation, and (2) compare the drought responses of two purple and red skin potato genotypes within the *Andigena* group. The present study will provide results and information regarding the use of spectral indexes related with physiological variables in potato to identify drought resistance genotypes and develop a useful tool for plant phenotyping.

## Materials and methods

### Plant material

Two potato genotypes belonging to the *Solanum tuberosum* L. group *andigena* were used in this study: Diacol Capiro (also known as R12) and Perla Negra (CIP code N° 391691-96 and called Serranita in Peru), both have semi-erect growth habit. Diacol Capiro is the most widely cultivated variety for fresh markets and for the industry in Colombia. The tuber skin is predominantly red with purple as a secondary color, and the flesh color is cream. Perla Negra was a recently introduced material from the International Potato Center CIP under the international project “Innovation and Development Network” toward the efficient dissemination and pro-poor impact mechanisms with new potato varieties in the Andean zone. Specifically, Perla Negra has a purple skin and a pale yellow flesh and its high content of anthocyanin will be an indicator of potential drought tolerance.

### Experimental conditions

The experiments were conducted at the facilities of Corporacion Colombiana de Investigación Agropecuaria (CORPOICA) in the research center C. I. Tibaitatá (14 Km via Mosquera, Colombia, 4°41′45”N and 74°12′12”W), from the end of June to November 2015. The trials were carried out in a glasshouse with controlled conditions. The minimum and maximum daily temperatures were 14.04 and 19.32°C, respectively, and the atmospheric relative humidity varied between 48.3 and 82.4%. The daily average solar radiation was 7.4, 7.0, 6.9, and 6.8 MJm2^*^d^−1^ in August, September, October and November, respectively (WatchDog 2900ET Weather Station, Aurora, IL).

Tubers were planted in pots (20 L) filled with 14 kg of soil (5.36 pH, 0.40 ds.m^−1^, 14.23% OM). Each pot was fertilized with 40 g of Triple-15 (15% N, 15% P and 15% K) and 5 g of Sulfomag® per seedling. Additionally, nutrient solutions were added 15, 21, and 42 days after emergence (dae) following the methodology reported by Mateus (2010).

Soil moisture was controlled using a tensiometer (Soilmoisture Equipment Corp, USA) and the watering of each pot was carried out with a test tube according to the tensiometer‘s readings. The soil water potential in the control did not decline bellow −0.03 MPa. Three treatments were applied to each genotype: a control treatment in which the substrate was maintained at field capacity (soil water potential did not decline below −0.03 MPa), and two water restriction treatments that started 10 and 15 days from tuber initiation (60 dae) (Table 1). Finally, after treatments were applied plants were well watered until harvest.

**Table 1:** Treatments used to evaluate drought response in two potato genotypes.

**Code**	**Treatments**
Cr	Perla Negra control
C10	Perla Negra 10 days of drought
C15	Perla Negra 15 days of drought
Dr	Diacol control
D10	Diacol 10 days of drought
D15	Diacol 15 days of drought

*Drought was applied 60 dae*.

A randomized complete block design was used with four replicates and six plants per treatment. Four destructive samplings were carried out (4 plants per treatment) before water restriction was initiated (60 dae), at the end of each water restriction treatment (70 and 75 dae) and after watering (95 dae).

### Gas exchange

Physiological measurements were made on expanded and sun-exposed leaves located in the middle third section of the plant. Gas exchange was measured using a portable photosynthesis system CIRAS 3 (PP Systems, USA) under ambient light (cuvette details: area 1.5 cm^2^, CO_2_ 400 ppm, temperature 22°C, cuvette flow rate 300 cc min-1 and relative humidity 50%), obtaining values for net photosynthesis (A), stomatal conductance (g_s_), internal leaf CO_2_ (Ci), vapor pressure deficit (VPD), transpiration (E) and water-use efficiency (WUE = A.E^−1^), the later according to Hunt (1982), and following the user manual version 1.06. All measurements were taken from 11:00 to 13:00 ensuring the high light intensity. It is based on a previous characterization during 8 days measuring gas exchange each hour since 8:00 to 16:00 (data not shown). The ceptometer and CIRAS3 have their own PAR sensor.

### Chlorophyll concentration, water content, and leaf area index

Chlorophyll concentration (ChlSPAD) was estimated using a portable chlorophyll content meter CCM-200 (Opti-Sciences, USA). The data collected corresponded to the green color content of a leaf, and its value was equivalent to the amount of light transmitted by the leaf in two regions of the red and infrared wavelengths. The amount of red light absorbed indicated the quantity of chlorophyll, whereas the quantity of light absorbed next to the infrared wavelength was used as an internal reference to compensate for leaf thickness (Torres Netto et al., 2005). The leaf area index (LAI) was estimated using an Accupar LP-80 ceptometer (Decagon Devices, USA). Due to semi-erect growth habit of Perla Negra and Diacol Capiro, plants were tutored (45 dae) and separated avoiding overlapping between them. Additionally, the analysis where based on segments involved reducing the error based on clumping effects of a canopy. Similar studies on single plants have estimated LAI in greenhouse under similar conditions. (Mushagalusa et al., 2008; Moeller et al., 2014; Mendoza-Pérez et al., 2017). Finally, three readings per plant were averaged to get the final LAI value. Total water content (H_2_Ot) was evaluated by difference of dry and fresh mass.

### Yield

Tuber harvest was carried out 130 dae for Perla Negra and 140 dae for Diacol Capiro according to the production cycle of each genotype and following the cultural classification practice used in Colombian productive potato areas. Tubers were then classified into four categories as follows: 1, 2, 3 and “Richie,” where large tubers were included in category 1 and the smallest ones were classified as “Richie.” The final harvest occurred when plants in each irrigation treatment reached senescence onset. Fresh and dry mass was measured. Stems, leaves and roots were weighted with a digital balance (Ohaus sp4001- USA). These samples were dried at 80°C during 3 days. Tubers were cut and dried for 24 h at 120°C and 80°C for 3 days.

### Canopy spectral information

The reflectance information was captured using of spectroradiometer FieldSpec® 4 Hi-Res (ASD Inc., USA) from 350 to 2,500 nm with interval of 1 nm. A leaf clip was used for nondestructive data collection. The leaf clip and contact probe device reduces interferences, such as atmospheric disturbances (e.g., sunlight) due to a trigger lock/release gripping system and a self-contained light source. The integrated pre-calibrated white reference panel of the ASD leaf clip was used for calibration purposes each 20 measurements (Lehmann et al., 2015). Those were taken (25, 45, 56, 62, 63, 67, 68, 69, and 107 dae.) on expanded and sun-exposed leaves located in the middle third section of the plant at morning (~9 am). For each plant sampled, six measurements on completely extended leaflets were carried out. Two plants per treatment in each block were evaluated for a total of eight plants per treatment. The spectral signatures and indexes were calculated using custom python script. The indexes were calculated in the spectral region between 526 and 970 nm (Bowman et al., 2015; Table 2). The visible spectrum ranges from 526 to 780 nm and the near infrared (NIR) extends from 780 nm to 970 nm. Those indexes were correlated with sixteen physiological variables measured (LAI, Ci, gs, VPD, A, E, WUE, SPAD, H_2_Ot, yield, FA, NAR, RGR, LAR, EAR, and RGR_tub_).

**Table 2:** Indexes and formulas derived from reflectance information.

Reflectance index^*^		Formula^**^	**References**
NDVI	Normalized Vegetation Index	((R(900 nm)−R(680 nm))/(R(900 nm)+R(680 nm))	Rouse et al., 1973
SR	Simple Ratio	(R(900 nm)−(R(680 nm))	Jordan, 1969
RNDVI	Normalized Difference Vegetation Index	(R(900 nm)−R(680 nm))/(R(900 nm)+R(680 nm))	Raun et al., 2001
PRI	Photochemical Reflectance Index	(R(531 nm)−R(570 nm))/(R(531 nm)+R(570 nm))	Penuelas et al., 1997
PSSRa	Pigment-Specific Simple Ratio of Chl-a	(R(800 nm))/(R(680 nm))	Blackburn, 1999
WI	Reflectance Water Index	(R(900 nm))/(R(970 nm))	Penuelas et al., 1993
NWI1	Normalized Water Index 1	(R(970 nm)−(900 nm))/(R(970 nm)+R(900 nm))	Babar et al., 2006b; Prasad et al., 2007
NWI2	Normalized Water Index 2	(R(970nm)−R(850nm))/(R(970nm)+R(850nm))	
NWI3	Normalized Water Index 3	(R(970 nm)−R(920 nm))/(R(970 nm)+R(920 nm))	
NWI4	Normalized Water Index 4	(R(970 nm)−R(880 nm))/(R(970 nm)+R(880 nm))	
ONI	Oryza N Index	(R(810 nm))/(R(560 nm))	Xue et al., 2004
DZNI	Dry Zea N Index	(R(575 nm))/(R(526 nm))	Zhao et al., 2003

*The spectral region used ranged between 526 and 970 nm. Chlorophyll's electromagnetic absorption range is between 400 and 500 nm, and from 600 to 700 nm*.

* *Reflectance indexes taken from Bowman et al. (2015)*.

** *R represents the reflectance measured at a particular wavelength*.

Growth parameters were calculated according to Hunt (1982): net assimilation rate (NAR), relative growth rate (RGR), leaf area ratio (LAR), economic assimilation rate (EAR) and relative growth rate for tubers (RGR_tub_). In addition, foliar area (FA) was measured with the CompuEye software (Bakr, 2005) where the leaf area is calculated based on a leaf image of VGA size. Images have background contrasting with that of the leaves. It was marked with two points which length between them was 20 cm.

### Statistical analysis

The SAS v9.3 software (SAS Institute, Cary, NC, USA) was used to carry out an ANOVA and a GLM (General Lineal Model) procedure, to evaluate the effects of treatments on physiological variables using a Tukey test with repeated measures over time. Significance on statistical tests was assessed at *P* < 0.05. The relationship between the reflectance indexes and physiological variables were tested with Pearson‘s Correlation Coefficients.

## Results

### Gas exchange

**Figure 1:**
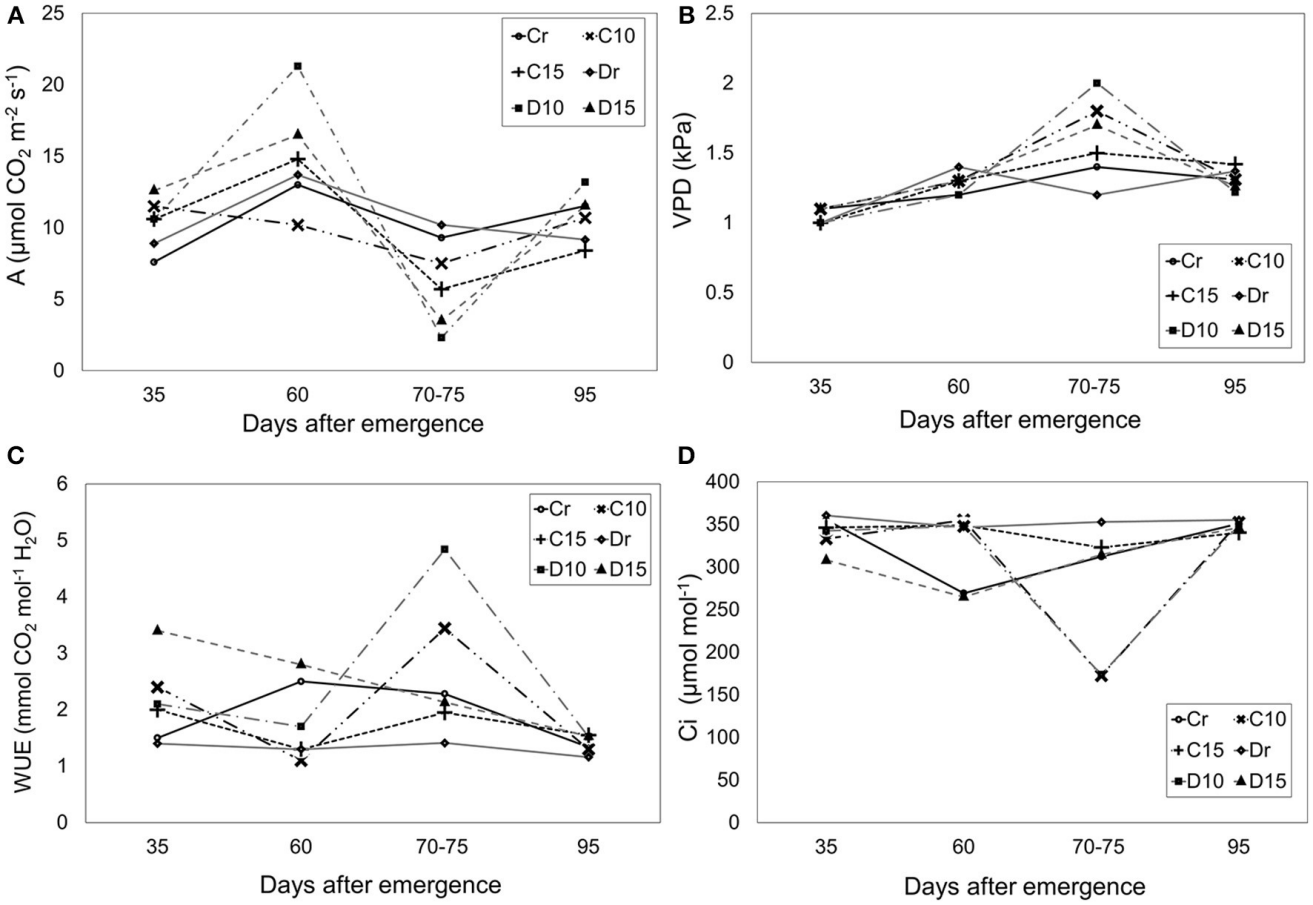
Physiological traits measured under three drought treatments [10 and 15 days without water and a control (r)] in two genotypes: Perla Negra = C and Diacol Capiro = D. Photosynthesis **(A)**, **(B)** Vapor pressure deficit (VDP), **(C)** Water use-efficiency (WUE), and **(D)** Intra cellular carbon dioxide (Ci). Significant difference was found for D10 (VPD) (Dunnett comparison at 0.05).

**Figure 2:**
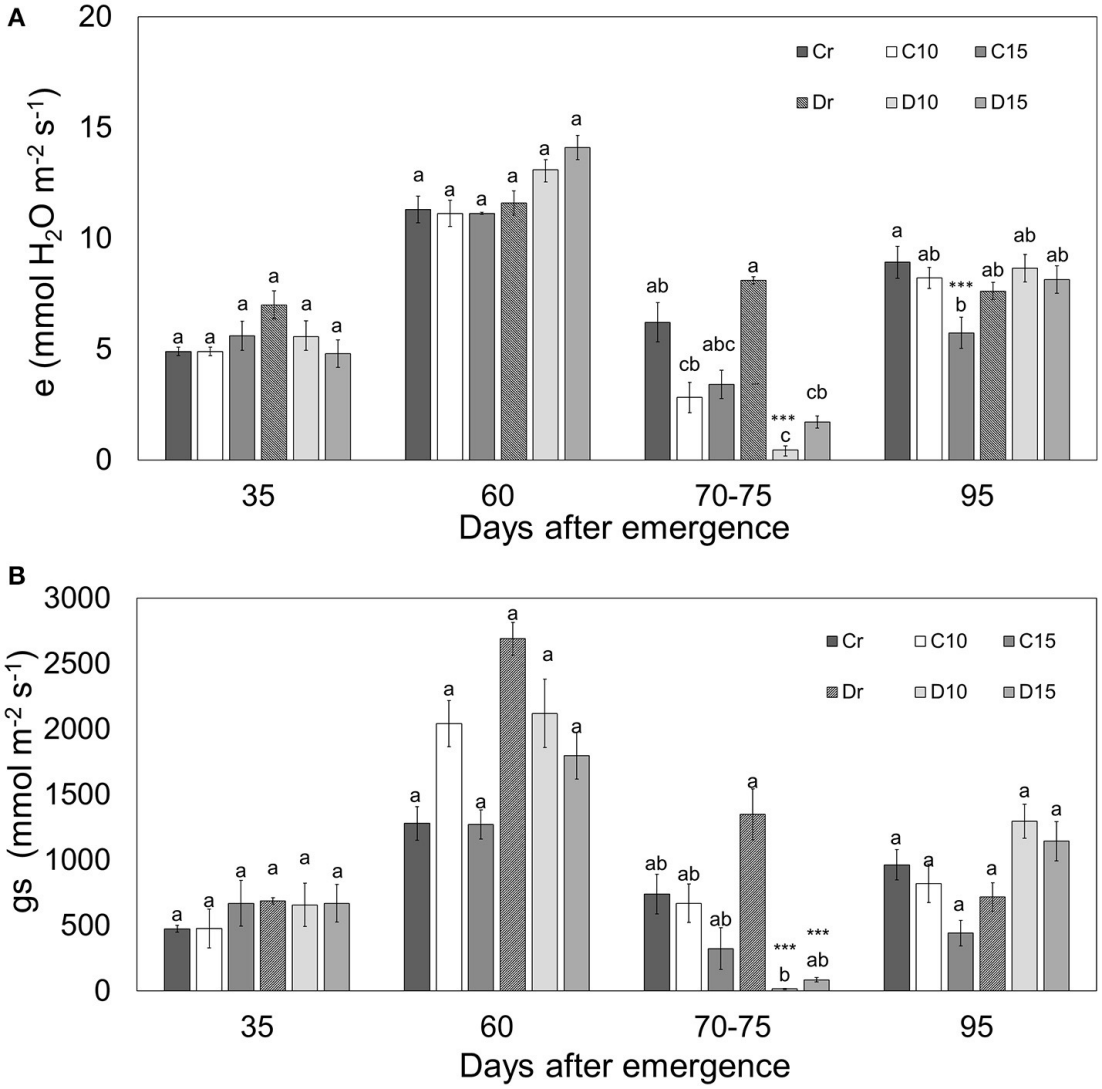
Transpiration (e) **(A)** and stomatal conductance (gs) **(B)** in two genotypes (Perla Negra—C and Diacol Capiro—D) under three drought treatments [10 and 15 days without water, and a control (r)]. Means with the same letter are not found significantly different by Tukey's test. Dunnett comparisons are significant at 0.05 (^***^).

All treatments presented similar levels of photosynthesis (~10.60 μmol CO_2_ m^−2^ s^−1^), VPD (~1.31 kPa), water use efficiency (WUE) (~2.1 mmol CO_2_ mol^−1^ H_2_O) and internal carbon dioxide (~321.79 μmol mol^−1^) without significant differences (Figure 1). However, a reduction in photosynthesis was observed at 70–75 dae. During drought treatments, photosynthesis decreased for C10, C15, D10 and D15 in a ratio of 19, 39, 77, and 66%, respectively. In addition, treatments under 10 days of drought (70–75 dae) presented the lowest Ci values (Figure 1D) with decreasing ratios (44 and 50% for C10 and D10, respectively). In contrast, Ci values were higher in Perla Negra under 15 days of drought (3%) while in Diacol Capiro these decreased to 10%. Similarly, VPD values increased under drought conditions (70–75 dae) for C10 (28%), C15 (7%), D10 (66%), and D15 (41%) (Figure 1B). Moreover, during drought periods Diacol Capiro's control (Dr) showed the lowest WUE value, whereas plants of both genotypes under 10 days of drought (D10 and C10) presented higher values because the photosynthesis/transpiration relation was greater than in other treatments (Figures 1A, 2B). D10 showed the highest WUE value after a drought period (Figure 1C).

The stomatal conductance had a direct effect on transpiration during water deficit situations. As was expected, there were no significant differences for transpiration (e) and stomatal conductance (g_s_) between 35 and 60 dae, because all treatments received the same amount of water. Transpiration displayed maximum values of ~11 mmol.m^−2^.s^−1^ in well hydrated plants of the two genotypes tested (Figure 2). However, Diacol Capiro presented a reduced transpiration level under both drought treatments (70–75 dae) for D10 (0.46 mmol m^−2^ s^−1^) and D15 (1.7 mmol m^−2^ s^−1^). Therefore, Diacol Capiro plants displayed reductions for D10 (98%) and D15 (78%) in relation to its control (Dr). Similarly, stomatal conductance decreased under drought for D10 (98%) and D15 (93%). Interestingly, Perla Negra did not show significant differences under water stress conditions, however, at the final stage (95 dae), its transpiration was significantly low (64%).

### Chlorophyll concentration, water content, and leaf area index

**Figure 3:**
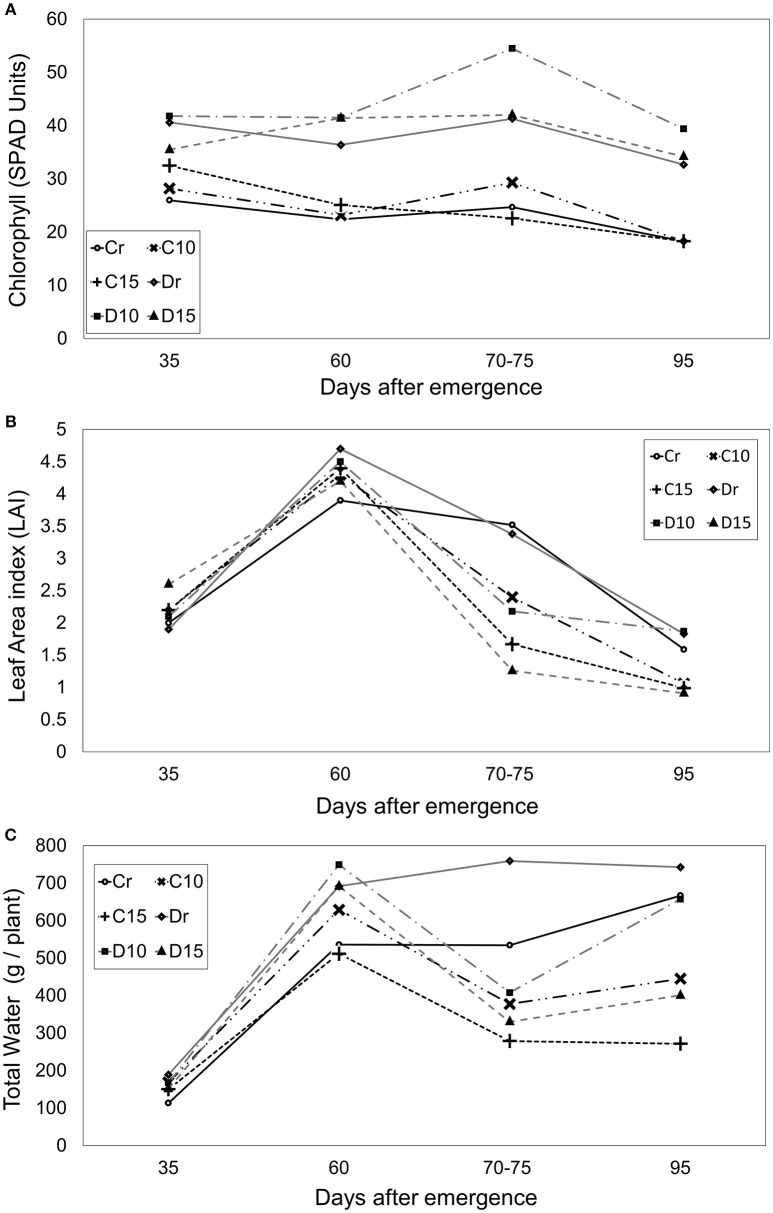
Chlorophyll concentration **(A)**, leaf area index **(B)** and total water **(C)** during different crop stages (days after emergence - dae) in two genotypes (Perla Negra—C and Diacol Capiro—D**)** under three drought treatments [10 and 15 days without water, and a control (r)].

There were significant differences among genotypes, showing Diacol Capiro an average of 41 SPAD and Perla Negra an average of only 24 SPAD. Therefore, Diacol Capiro displayed greater chlorophyll content (67%) than Perla Negra, and in general, the results showed increased chlorophyll content values under water stress conditions (Figure 3). However, there were no significant differences among treatments. Nevertheless, under 10 days of water stress, the chlorophyll value increased for D10 (31%) and C10 (18%), compared with their controls (Dr and Cr). Interestingly, D15 decreased 2% and C15 increased 8% SPAD units (Figure 3A).

The maximum LAI expression was presented at start flowering (60 dae) where Diacol Capiro was 6% higher than Perla Negra with values of 4.7 and 4.2, respectively. During drought period (70–75 dae), Diacol Capiro presented a lower leaf area index (LAI) value (4%) than Perla Negra under normal watering conditions. Plants under drought stress, LAI presented significant differences between treatments. Therefore, the controls (Dr and Cr) showed the highest values during drought stress period compared with other treatments that presented a reduced LAI, e.g., D10 (35%), D15 (62%), C10 (31%), and C15 (52%). As expected, during the rehydration phase (95 dae), LAI became stable without differences between treatments (Figure 3B).

Diacol Capiro showed higher water content (42%) than Perla Negra under good watering conditions. However, when plants were submitted to water stress, a significant decrease in total water content was observed (Figure 3C). Particularly, reduction in 47, 46, and 56% were found for C15, D10 and D15, respectively. Interestingly, C15 was the treatment that showed the lowest total water content value during and after the drought stage.

### Yield

**Figure 4:**
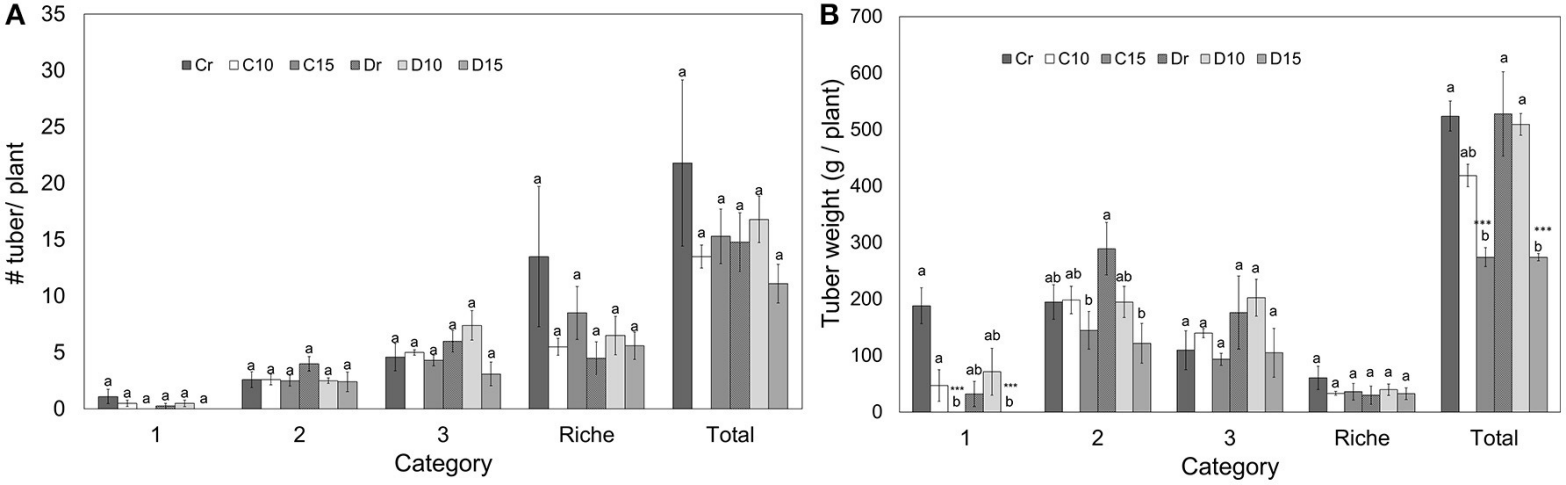
Number of tubers per plant **(A)** and tuber weight **(B)** classified into categories that are found in two genotypes (Perla Negra—C and Diacol Capiro—D) under three drought treatments [10 and 15 days without water, and a control (r)]. Tukey means with the same letter are not significantly different. Dunnett comparisons are significant at 0.05

On average, one well-watered potato plant produced 22 tubers weighing in total 524 g for Perla Negra and Diacol Capiro produced 15 tubers that weighed 528 g. Considering all treatments, the number of tubers per category did not show significant differences (Figure 4A). However, Perla Negra (Cr) had the highest number of tubers classified in category “richie” showing no significant differences in weight compared with other treatments, which means that the tubers produced were very small. In addition, tuber weight per plant was affected under 15 days of drought for both genotypes, since no tubers obtained could be classified in category 1 (Figure 4B). Similarly, Diacol Capiro's tubers from D15 plants belonging to category 2 were significantly lower (58%) compared to its watered control (Dr). In contrast, Perla Negra plants C15 presented a slight reduction of 25% in its yield. Finally, under 15 days of stress both genotypes showed a similar yield reduction, i.e., 47% in Perla Negra and 48% in Diacol Capiro. Therefore, under these experimental conditions Perla Negra showed less affectation of its tuber production than Diacol Capiro, and this material has to be further evaluated on field drought trials.

### Canopy spectral information

**Figure 5:**
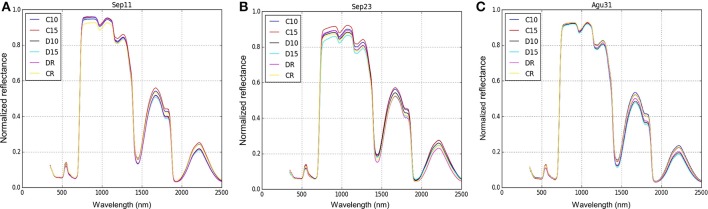
Spectral signature profile of two genotypes (Perla Negra—C and Diacol Capiro—D) under three drought levels. The spectral data were collected in three different periods: before drought **(A)**, 9 days of suffering from drought **(B)** and after drought—rehydration **(C)**.

The spectral profiles were plotted showing a clear variation between signatures from 700 to 1,300 nm. Moreover, comparing the spectral signature before (Figure 5A), during (Figure 5B) and after (Figure 5C) the drought treatment, there was a clear difference between D15 (lowest reflectance) and C15 (highest reflectance).

The results showed that spectral indexes presented correlation with LAI, H_2_Ot, FA, LAR, and RGR_TUB_ (Table 3). Specifically, water indexes (NWI1, NWI2, y NWI4) were negatively correlated with all physiological variables except LAR. In contrast, indexes WI, NDVI, RNDVI, and DZNI showed positive correlation with H_2_Ot. In addition, NWI3 was negatively correlated with H_2_Ot. Interestingly, half of the reflectance indexes evaluated showed correlation with RGR_TUB_, i.e., the plants efficiency to produce dry tuber mass. Also, PRI presented the highest correlation coefficient with RGR_TUB._

**Table 3:** Pearson's correlation between reflectance indexes and physiological variables.

**Physiological variables**	**Reflectance indexes**											
	**wi**	**nwi1**	**nwi2**	**nwi3**	**nwi4**	**sr**	**rndvi**	**ndvi**	**pri**	**pssra**	**oni**	**dzni**
LAI	0.27921	−0.62158	−**0.85564**	−0.52771	−**0.71344**	**0.73109**	0.38956	0.37412	**0.93215**	**0.74756**	0.62348	0.19029
	0.1864	0.0012	**<0.0001**	0.0080	**<0.0001**	**<0.0001**	0.0599	0.0717	**<0.0001**	**<0.0001**	0.0011	0.3731
H_2_Ot	**0.79672**	−**0.84996**	−0.66592	−**0.87514**	−**0.80409**	0.63429	**0.79623**	**0.79566**	0.52364	0.62531	**0.75680**	**0.77678**
	**<0.0001**	**<0.0001**	0.0004	**<0.0001**	**<0.0001**	0.0009	**<0.0001**	**<0.0001**	0.0086	0.0011	**<0.0001**	**<0.0001**
FA	0.53013	−**0.76562**	−**0.86824**	−0.70811	−**0.81467**	**0.79601**	0.61131	0.59949	**0.87593**	**0.80413**	**0.74809**	0.45990
	0.0077	**<0.0001**	**<0.0001**	0.0001	**<0.0001**	**<0.0001**	0.0015	0.0020	**<0.0001**	**<0.0001**	**<0.0001**	0.0238
LAR	0.50722	−0.58176	−0.73292	−0.48779	−0.64847	**0.89306**	0.77449	0.75307	**0.86168**	**0.89115**	0.78420	0.12556
	0.0317	0.0113	0.0005	0.0400	0.0036	**<0.0001**	0.0002	0.0003	**<0.0001**	**<0.0001**	0.0001	0.6196
RGR_tub_	0.32447	−0.77680	−**0.88840**	−0.69747	−**0.83101**	**0.81788**	0.62149	0.58668	**0.91621**	**0.82648**	0.73310	−0.06231
	0.1890	0.0001	**<0.0001**	0.0013	**<0.0001**	**<0.0001**	0.0059	0.0105	**<0.0001**	**<0.0001**	0.0005	0.8060
Pearson Correlation Coefficients				Prob > |r| under H0: Rho = 0								

*The significant values are highlighted in bold*.

## Discussion

### Gas exchange

In the present study, the photosynthesis (A) decreased during the period of 10 and 15 days of water stress without big differences compared to the watered control. However, Diacol Capiro plants were the most affected under stress conditions. Moreover, similar reduction of internal leaf CO_2_ (Ci) was found in C10 and D10. At severe stress states, C15 showed Ci stability compared to the control, while Diacol Capiro maintained its decreasing pattern. This phenomenon is mainly due to photosynthesis decrease resulting from a reduction of internal carbon, where the biochemical capacity for carbon assimilation and utilization is reduced during drought periods (Reddy et al., 2004; Oliver et al., 2009). Thus, decreased Ci confirms the predominance of stomatal limitation in restricting photosynthetic rate in the early water loss phase (Flexas and Medrano, 2002). During stomata closure, the CO_2_ inside the leaf (Ci) initially declines with increasing stress and then increases as drought becomes more severe (Lawlor, 1995). However, the debate continues as to whether drought mainly limits photosynthesis through stomatal closure or through metabolic impairment (Tezara et al., 1999; Lawson et al., 2003). In fact, under stress conditions that limit CO_2_ fixation, the rate of production of reducing power is greater than the rate of its use by the Calvin cycle. Protection mechanisms against excess in reducing production power are thus an important strategy under water stress (Reddy et al., 2004). These photoprotective mechanisms compete with photochemistry for the absorbed energy, leading to a decrease in the PSII's quantum yield (Genty et al., 1989). The photosynthesis rate in higher plants depends on the activity of ribulose-1, 5-bisphosphate carboxylase/oxygenase (Rubisco) as well as on the synthesis of RuBP (Chaitanya et al., 2002). Decreasing synthesis of Rubisco under drought was evidenced by a rapid decrease in the abundance of small subunits of Rubisco (rbcS) in tomato (Vu et al., 1999). Finally, our results were consistent with the general findings of Vos and Haverkort (2007) where the net photosynthesis of potatoes is not easily affected by drought stress and may only be affected under severe water stresses.

The VPD of the atmosphere can negatively affect plant growth as plants reduce stomatal conductance for water vapor (g_wv_) in response to increasing VPD, limiting the ability of plants to assimilate carbon (Ocheltree et al., 2013). In our study, the greatest variations were viewed for Diacol Capiro with (Figure 1) and Perla Negra showed a more stable VPD. Similar results were presented by Silva et al. (2013), where the intensification of VPD is related directly with higher stomatal closure and correlated with photosynthesis reduction. Therefore, a change of VPD on the net assimilation rate indicates a direct effect of VPD on stomata (James et al., 1983). High VPD may decrease photosynthetic performance due to stomatal (diffusion) and nonstomatal (mesophyll) limitations (Shibuya et al., 2016). Most plants show a nonlinear, asymptotic transpiration (E) response to increasing VPD, but some plants show a reduction in E as VPD continues to increase, which has been referred to as an “apparent feedforward” response (Monteith, 1995). The stomatal conductance sensitivity to VPD is a key regulator of water use and carbon assimilation strategies in plants (Ocheltree et al., 2013).

The increasing worldwide water resources shortage requires irrigation management optimization in order to improve water use efficiency (WUE) (Liu et al., 2006). In our study, the WUE increased with decreasing irrigation amount with values of 4.84 mmol CO_2_ mol^−1^ H_2_O that is consistent with previous studies of WUE in potato (Vos and Groenwold, 1989; Trebejo and Midmore, 1990; Onder et al., 2005; Fleisher et al., 2008). WUE has been known to increase with increasing drought stress and reduced water supply (Meyers et al., 1984). However, superior water-use efficiency (WUE) is not synonymous with drought resistance and high yield under drought stress (Blum, 2009). Moreover, drought tolerance was found to be associated with low WUE when analyzed by delta (carbon isotope discrimination C12/C13) under limited water supply (Solomon and Labuschagne, 2004). For instance, our results suggested that Perla Negra could be drought resistant because its WUE values were lower than the ones of Diacol Capiro under water stress conditions. Similarly, Pinheiro et al. (2005) found that a drought resistant *Coffea canephora* clone had relatively low WUE. Nevertheless, further studies using Perla Negra under severe water deficit on field, including soil management practices (e.g., plant nutrition) are necessary.

Under water stress conditions Diacol Capiro presented a reduced stomatal conductance (g_s_) and transpiration (*e*). Similar results were reported by Fleisher et al. (2008) where transpiration rates were lower for plants grown under water stress treatments. Interestingly, Perla Negra did not show significant differences for g_s_ or *e*. During drought stress, stomatal closure inhibits transpiration more than it decreases intercellular CO_2_ concentrations (Taiz and Zeiger, 2002), helping to conserve water and maintaining an adequate leaf water status, but at the same time, reducing g_s_ concentrations (Chaves et al., 2002). Furthermore, the stomatal conductance began to have a greater effect on transpiration than the VPD (leaf-air) during water deficit (Silva et al., 2013).

Drought stress progressively decreases CO_2_ assimilation rates due to reduced stomatal conductance. A good correlation between leaf water potential and stomatal conductance always exists, and it should be taken as an integrative parameter to assess photosynthetic rate, even under drought stress (Reddy et al., 2004). In contrast, negative correlation has been found with g_s_ and *e* compared with WUE (Blum, 2009). Therefore, genotypes that produce the same amount of photoassimilates while their *e* decreases could potentially be drought tolerant materials. Consequently, it is possible to enhance drought tolerance by reducing transpiration (Bañon et al., 2006). As long as the biochemical pathways of photosynthesis continue to be (apparently) unresponsive to conventional breeding schemes, genotypic transpiration efficiency and WUE are mainly driven by plant traits that reduce transpiration and crop water-use, processes which are of utmost importance for plant production (Blum, 2009).

### Chlorophyll concentration, water content, and leaf area index

Under water stress conditions the chloroplast concentration increases due to loss of turgor and the chlorophyll's photochemical activity is reduced (Genc et al., 2013). However, chlorophyll content also depends on the growth stage. In fact, our results reported a decreasing behavior during the final phase (95 dae) and seen by finding chlorotic leaves during the harvest phase (Palaniswami and Peter, 2008). Similarly, this was observed in maize where chlorophyll readings decrease as the plant approaches maturity (Genc et al., 2013). In addition, our results showed that leaf area decreased under water stress causing chlorophyll concentration increases in plants submitted to slight water deficit. These results agree with those obtained by Teixeira and Pereira (2007), where leaf chlorophyll content increased significantly in response to drought (5.6%). According to Deblonde and Ledent (2001), the LAI reduction can affect the production of photoassimilates, dry matter and also tuber yield. Therefore, LAI is used to predict primary photosynthetic production, evapotranspiration and also as a reference tool for crop growth. Similar values of LAI in potato under greenhouse were found by Flores-López et al. (2016a,b) These results showed that Perla Negra is more photosynthetic efficient than Diacol Capiro because even though it had a lower LAI value at 60 dae, it has a greater yield that Diacol Capiro.

On the other hand, potato responds to drought stress with epinasty or with the leaves showing a downward curvature. Unlike wilting, epinasty does not involve turgor loss (Taiz and Zeiger, 2002). Epinasty avoids losing water when the leaf area and the leaf area index decreases. When potato plants suffer longer drought periods their leaves drop, and this is followed by wilting that starts from the lower strata leaves. Nevertheless, if leaves have some greenness, potato plants will recover turgor and finalize their production cycle. As expected in this study, the water content and LAI decreased proportionally to the severity of the drought stress on the genotypes assessed. Total water content of D10 and C10 had lower water content than the control plants, but C15 and D15 showed the lowest values during water stress periods (70 and 75 dae). Water tissue content affects physiological processes as stomatal conductance in several plant species under drought conditions; this is most probably due to increased sensitivity to xylem-carried ABA which is induced by low leaf water potentials (Wilkinson and Davies, 2002).

### Yield

In this study, low water availability affected tuber yield. This is one of the most important variables to evaluate drought tolerance in potato plants, and it is very well known that under field conditions, drought causes drastic losses in potato tuber yield and/or quality (Deblonde and Ledent, 2001; Walworth and Carling, 2002; Stark et al., 2013). Consequently, drought evaluation is based on a combination of yield stability and high relative yield under water deficits (Obidiegwu et al., 2015). The magnitude of the drought effects on potato production depends on the phenological timing, duration, and severity of the stress (Schafleitner, 2009). For instance, Dalla-Costa et al. (1997) showed that tuber yield reduction under stress treatments was almost proportional to biomass decrease, and was a result of reduced leaf area and less photosynthesis per leaf area unit. For this reason, water use efficiency is calculated as the proportion of fresh tuber yield and the water content applied (Trebejo and Midmore, 1990).

Our results suggest that yield reduction under water stress can be caused by reduced leaf area and/or reduced photosynthesis per leaf area unit. Therefore, facing the current climate change scenario, these parameters have to be considered on every potato breeding program where ideally all potato cultivars should be drought tolerant with a high yield potential under drought stress (Soltys-Kalina et al., 2016).

### Canopy spectral information

Five physiological variables were correlated with spectral indexes. Specifically, some vegetative and photochemical indexes (SR, PRI, PSSRa) were positively correlated with mayor physiological variables, except H_2_Ot. Previously, these indexes have been reported as biomass indicators (Babar et al., 2006a). Likewise, water indexes or indexes using the 970 nm wavelength (minor water absorption band) have been used as plant water status indicators (Bowman et al., 2015). The spectral data showed that reflectance at water sensitive wavebands (940–970 nm) before irrigation was slightly higher than after irrigation. Similar results were reported by Genc et al. (2013) evaluating spectral reflectance of sweet corn under water stress. Reflectance properties of plants depend in part on the amount of water stored in the leaf cells, in particular in the near infrared (NIR) region (Rodríguez-Pérez et al., 2007; Govender et al., 2009). Crop yield is based on a complex interaction of different agronomic factors such as density, vigor, maturity and stress resistance that altogether, can be used as yield indicators. However, the spectral reflectance of the crop is dependent on the phenology, stage type and crop health. For instance, early prediction of potato yield using multispectral images was reported by Al-Gaadi et al. (2016). Their results suggest that the PRI index will be a useful parameter to predict yield.Similarly, the PRI_512_ index has been reported as an indicator of water stress (Hernández-Clemente et al., 2011). In addition, reflectance measured before irrigation was generally higher than after irrigation in the NIR region, and a similar reflectance behavior under water stress was found in corn plants (Genc et al., 2013).

A spectral model in the form of a normalized difference index was identified as an example for nondestructive estimation of RWC (Zygielbaum, 2009). This suggests that spectral information will be a useful tool to predicting yield in potato, including plants under water stress. Previous studies on wheat presented correlation between vegetative and photochemical related indexes with biomass production and yield (Babar et al., 2006b; Prasad et al., 2007; Gutierrez et al., 2010; Bowman et al., 2015). In our study, SR, PRI, and PSSRa had correlation with biomass production and LAI, LAR, FA and RGRtub; RNDVI, NDVI were correlated with total water content. Hence, the use of spectral indexes could be useful to get physiological indicators without using destructive sampling. Thus, spectral reflectance is a potential high-throughput method for assessing abiotic and biotic stresses in potato. However, further studies have to be done to standardize indexes and prediction models to determine water stress on field trials.

## Conclusion

Based on physiological evaluations, Perla Negra was less affected under water stress conditions than Diacol Capiro. Therefore, Perla Negra's photoassimilate production was constant even though plants were under drought stress. Interestingly, during the hydration phase to field capacity, transpiration of C15 was significantly low, and it may have been caused by a senescence growing cycle that is shorter than the one in Diacol Capiro. This drought tolerant genotype has to be evaluated at population level (half-sib) to evaluate heritability and possible breeding gain within our breeding program.

These results confirm the potential use of spectral reflectance indexes on general plant phenotyping and particularly regarding drought tolerance. These indexes will be useful to accelerate the screening of genebank germplasm to select the putative drought tolerant parents and select the segregant population within a breeding program. The goal is to evaluate a diverse set of germplasm in multienvironmental trials to elucidate the relationship between drought response and genotype × environment interactions. Additionally, the Colombian potato germplasm bank has been characterized with high-dense SNP array (Berdugo et al., 2017). Therefore, the spectral data and genotyped diversity panel will provide information about loci involved on stress tolerance, and moreover, using genomic selection models the best genotypes with the highest genomic estimated breeding values (GEBV) can be predicted (Desta and Ortiz, 2014); this will altogether provide valuable tools for potato breeding programs.

## Author contributions

CG, RV, and AA conceived and conducted the trials. AR assisted in the data collection. CG, AA, and AR were primarily responsible for writing the manuscript.

### Conflict of interest statement

The authors declare that the research was conducted in the absence of any commercial or financial relationships that could be construed as a potential conflict of interest.
